# Hyperhomocysteinemia is related to large vessel occlusion in young patients with COVID‐19: Two case reports

**DOI:** 10.1002/ccr3.6716

**Published:** 2022-12-13

**Authors:** Seyedehnarges Tabatabaee, Fatemeh Rezania, Sevim Soleimani, Zahra Mirzaasgari

**Affiliations:** ^1^ Division of Neurology Firoozgar hospital, Iran University of Medical Sciences Tehran Iran; ^2^ Neurosciences Department St Vincent's hospital Melbourne Victoria Australia; ^3^ Student Research Committee, Faculty of medicine Shahid Beheshti University of Medical Sciences Tehran Iran

**Keywords:** COVID‐19, hyperhomocysteinemia, large vessel occlusion, young stroke

## Abstract

Here, we report two cases of previously healthy young men with COVID‐19 infection who developed acute ischemic stroke due to large vessel occlusion followed by secondary events concerning for a further thromboembolic event. We hypothesize that the hypercoagulable state related to COVID‐19 exacerbated the underlying hereditary thrombophilia.

## Graphical abstract

This report describes two adults with acute ischemic stroke due to large vessel occlusion after COVID‐19 infection. The COVID‐19 related hypercoagulable state may exacerbated the underlying thrombophilia due to MTHFR gene.

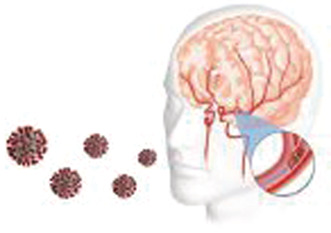

## 1 BACKGROUND

Numerous neurological complications of COVID‐19 have been identified. Severe infection with corona virus could result in headache, epilepsy, myasthenia gravis, cranial neuropathies, cerebrovascular events, and encephalitis.^1^, ^2^, ^3^, ^4^, ^5^, ^6^ Coagulopathy is a common feature of the disease, and it is associated with poor prognosis.^7^ The association between hyperhomocysteinemia and cerebrovascular disorders have been studied.^8^ Elevated plasma homocysteine levels could take place secondary to insufficient intake of vitamin B12, vitamin B6, and folic acid as well as a genetic predisposition. Mutations in the gene encoding the protein MTHFR are the most commonly known genetic risk factor for hyperhomocysteinemia. The most common MTHFR mutations are two single nucleotide polymorphisms (SNP), C677T, and A1298C. Both SNPs result in a decreased MTHFR activity, which may cause hyperhomocysteinemia.^9^, ^10^ Here we present two young Asian men with a potential—previously silent—hereditary thrombophilia who presented with COVID‐19 pneumonia and two consecutive thromboembolic events; that is, ischemic stroke and nonarthritic anterior ischemic optic neuropathy(NAION).

**FIGURE 1:**
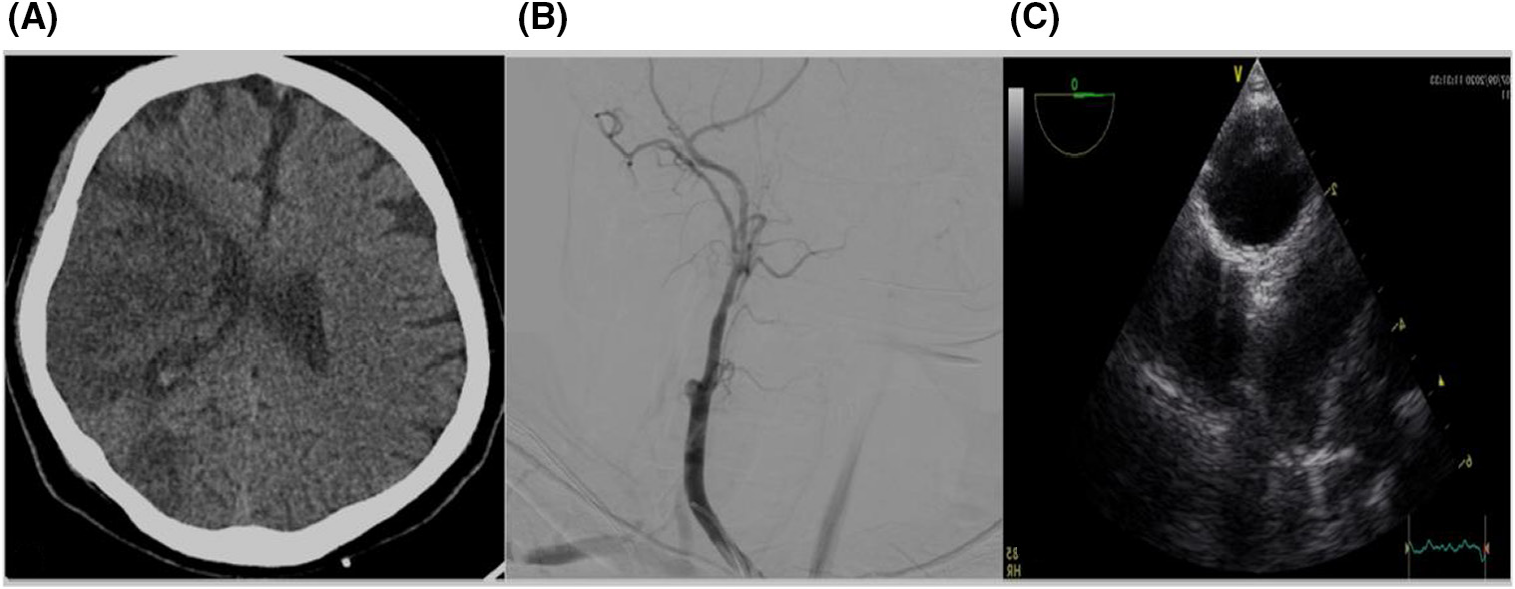
(A) CT‐scan, Hypodensity in MCA territory. (B) DSA, Rt ICA occlusion. (C) Trans thoracic echocardiography, a large aortic arch thrombus.

First case: A previously healthy 39‐year‐old Asian man was admitted to hospital with 10 days prodromal symptoms of fever, chills, malaise, and cough (Table 1). There was a positive family history for recurrent deep vein thrombosis in his mother and maternal aunt. The oropharyngeal swab test for coronavirus disease 2019 (COVID‐19) by qualitative real‐time reverse‐transcriptase–polymerase‐chain‐reaction (RT‐PCR) was positive. He was commenced on COVID‐19 treatment with Remdicivir as well as prophylactic anticoagulation. Despite this his respiratory symptoms were not improving. Ten days into his admission, he developed a left hemiparesis involving the face and limbs. He underwent emergent clot retrieval with recombinant tissue plasminogen activator (rTPA). His brain CT‐scan showed hypodensity within right middle cerebral artery (MCA) territory (Figure 1A). He was commenced on dual antiplatelets with Aspirin 100 mg daily and Clopidogrel 75 mg daily and atorvastatin 80 mg daily. He was intubated shortly after this due to altered conscious state together with severe respiratory involvement. Upon completion of another 7 days of treatment for COVID‐19 pneumonia with Remdicivir and Dexamethasone he was successfully extubated. Unfortunately, he declined further evaluation and left the hospital against medical advice at this point. He then represented few days later with a painless right eye vision loss upon waking in the morning. This presentation was about one month after his initial respiratory symptoms. He was transferred to our center to undergo diagnostic work‐up. He underwent carotid Doppler ultrasonography that showed right Internal carotid artery (ICA) complete occlusion. This was confirmed by digital subtraction angiography (DSA) that showed a complete occlusion of proximal right carotid artery (Figure 1B). His trans‐thoracic echocardiography revealed a large aortic arch thrombus (Figure 1C). He was commenced on anti‐thrombotic regime, initially with Heparin infusion. This was switched to Rivaroxaban 20 mg daily after. Dual antiplatelet therapy was ceased. A complete ophthalmic examination was performed by ophthalmologist who found a relative afferent pupillary defect (RAPD), a normal optic disc and impaired color vision (as examined by Ishihara color test) in the right eye. His visual acuity was reduced to hand motion in the right eye. Intraocular pressure, extraocular movements, and slit‐lamp examination were normal. His ophthalmic history was unremarkable. The occurrence of consecutive thromboembolic events in the absence of other risk factors except for COVID infection urged us to run an extensive thrombophilia screen. Homocysteine level was found to be elevated at 50 μmol/L (normal lab reference less than 15). Vitamin B12 and folic acid level were normal. MTHFR activity examined by genetic testing reveled a homozygous MTHFR A1298C variant. He continued on stroke preventive therapy with Rivaroxaban 20 mg daily and atorvastatin 80 mg daily, patient was discharged and lost to follow‐up.

**TABLE 1:** Clinical characteristics of two young patients presenting with LVO Stroke

Variable	Patient 1	Patient 2
Age‐years	39	34
Sex	Male	Male
Medical history and risk factor for stroke	None	None
Medications	None	None
NIHSS score	11	12
Outcome status	Discharged home	Discharged home
Signs and symptoms of stroke	Left hemiparesis, Dysarthria	Broca aphasia, Right hemiparesis
Vascular territory	Proximal right ICA	Proximal Left ICA
Imaging for diagnosis	CT, DSA	CT, MRI, Cervical color doppler sonography
Treatment for stroke	Heparin, Rivaroxaban	Heparin, Rivaroxaban
COVID‐19 symptoms	Malaise, Headache, Fever, Cough	Anosmia, Malaise, Cough
White‐cell count‐per mm3	9800	6800
Platelet count‐per mm3	183,000	263,000
Prothrombin time‐sec	14	13.5
Activated partial thromboplastin time‐sec	28	27
Fibrinogen‐mg/dl	323	255
D‐dimer‐ng/ml	Positive	Weakly positive

*Note*: Reference ranges are as follows: platelet count, 150,000–450,000 per cubic millimeter; prothrombin time, 12.3–14.9 s; activated partial‐thromboplastin time, 25.4–34.9 s; fibrinogen, 175–450 mg per deciliter. Scores on the National Institutes of Health Stroke Scale (NIHSS) range from 0 to 42, with higher numbers indicating more severe stroke.

## 2 SECOND CASE PRESENTATION

**FIGURE 2:**
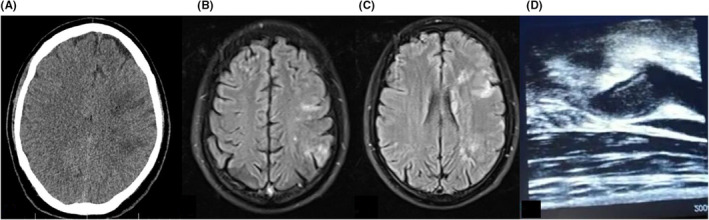
(A) CT. Scan, Hypodensity in MCA territory. (B, C) MRI, FLAIR, hyperintense lesion in MCA territory. (D) Carotid Doppler ultrasonography, acute left ICA complete occlusion.

A previously healthy 34‐year‐old, non‐smoker, Asian man was admitted to hospital with sudden onset right hemiparesis along with Broca's aphasia (Table 1). Twelve days prior, he had developed symptoms of anosmia, malaise, and cough. His oropharyngeal swab for COVID‐19 by qualitative RT‐PCR was positive. At his arrival, his brain CT‐scan showed hypodensity within Left anterior cerebral artery (ACA) and MCA territory (Figure 2A), and there was no time for clot retrieval treatment. His Magnetic Resonance Imaging of brain demonstrated a true diffusion restriction within ACA and MCA territory (Figure 2B,C). He was commenced on dual antiplatelet therapy, with Aspirin 100 mg daily and Clopidogrel 75 mg daily and Atorvastatin 80 mg daily. He then underwent carotid Doppler ultrasonography that showed an acute left ICA complete occlusion due to an intraluminal thrombosis of 5.9 × 9.8‐millimeter diameter (Figure 2D). His trans‐thoracic and transesophageal echocardiography were unremarkable. He was then commenced on anti‐thrombotic regime with Heparin infusion adjusted to 6‐hourly PTT monitoring. This was, however, changed to Rivaroxaban 20 mg daily. An acute rise in his serum creatinine level from 0.9 to 4.6 mg/dl was detected on Day 8. Although his acute kidney injury (AKI) suggested to be secondary to an acute tubular necrosis (ATN) due to Glomerulonephritis after renal consultation, kidney biopsy was not performed due to ongoing anticoagulation therapy to prove this. He received 1‐g intravenous methylprednisolone daily for 3 days. Kidney function subsequently improved. His Serum creatinine was 1.3 mg/dl at time of discharge. Kidney injury secondary to thromboembolic events, is one of the major complications of COVID‐19 and is considered as a mortality factor. The possibility of sequential thromboembolic events in the absence of other known risk factors except for COVID infection urged us to run an extensive thrombophilia screen. Homocysteine level was found to be 62 μmol/L. Therefore, MTHFR activity was examined by genetic testing and reveled a homozygous mutant for the MTHFR C677T polymorphism. Treatment continued with Rivaroxaban 20 mg daily and atorvastatin 80 mg daily, patient was discharged and lost to follow up.

## 3 DISCUSSION

Respiratory disorders have been reported as the main complication of COVID‐19. However, there are reports that documented many other symptoms such as cerebrovascular events especially secondary to an increase in risk of coagulopathy.^11^ This study describes two previously healthy men with COVID‐19 that presented with ischemic stroke due to large vessel occlusion (LVO) due to methylenetetrahydrofolate reductase (MTFHR) gene mutations. To the best of our knowledge, these are the first two cases describing MTFHR gene mutations in the setting of hypercoagulability and sequential thromboembolic events in COVID‐19 infection. The relative ratio of strokes in the young with age under 45 years old has been increasing over the past few decades.^12^ Hematologic and vasculopathic etiologies accounts for 44% of strokes in young patients.^13^ The most common causes in this age group are nonatherosclerotic, more often as a consequence of cardio embolism or arterial dissection.^14^ Inflammation‐driven hypercoagulable and vasculopathic state secondary to a recent infection is an independent risk factor for stroke (odds ratio 3.4–14.5), mostly respiratory in origin.^15^, ^16^ It has been reported that higher incidence of ischemic stroke is common with other respiratory viruses like Influenza and also with other coronaviruses^17^ and recent data suggests COVID‐19 confers a greater risk of stroke than influenza.^18^ Now it is well documented that COVID‐19 can lead to hypercoagulable state that results in venous and arterial thromboembolism.^11^, ^19^ Our patients were found to have elevated serum homocysteine, possibly contributing to their hypercoagulable states. Typically, a level 60 μmol/L is considered severely elevated.^20^ The elevated serum homocysteine which is the result of MTHFR gene polymorphisms as in case of MTHFR C677T could lead to decreased enzyme activity and therefore to an elevation of serum homocysteine level and in turn to thromboembolic events.^10^ It is controversial whether ischemic stroke is directly related to MTFHR mutation or not. In a recent study in Tunisian adults, it is confirmed that C677T and A1298C MTFHR variants are important risk factors for arterial ischemic strokes.^21^ In a meta‐analysis by Shan Kang et al MTHFR A1298C genetic polymorphism was associated with increased risk of ischemic strokes.^22^ Acute cerebrovascular disease was reported to occur 1.4% of COVID‐19 patients. The most common manifestation in 87.4% of these patients was with acute ischemic stroke.^23^ Mao et al reported 5.7% of patients with severe COVID‐19 infection developed cerebrovascular disease later in the course of illness.^24^ More recently, emergent large LVO has been reported in patients with COVID‐19. Shingo Kihira et al suggested that COVID‐19 is related to LVO rather than small vessel occlusion. In their study of 329 participants, 71 patients (21.6%) developed LVO.^25^ Likewise, in our cases carotid occlusion took place in previously healthy young adults. Several studies have found an association between hyperhomocysteinemia and NAION.^26^, ^27^ Different mechanisms have been suggested to play role. In our case, coagulation disorders could be in play.^28^ The high coagulopathic complications in our cases could be related to high levels of homocysteine along with MTHFR gene mutism that might have remained silent in the absence of COVID‐19. This highlights the importance of thrombophilia assessment in COVID‐19 patients with sequential events concerning for recurrent thromboembolism.

## 4 CONCLUSION

We presented two cases of previously healthy young men with COVID‐19 infection who developed acute ischemic stroke due to LVO followed by secondary events concerning for a further thromboembolic event. Both these patients were found to have hyperhomocysteinemia along with a MTFHR gene mutation. Hypercoagulable state as a result of COVID‐19 could have exacerbated the underlying silent coagulopathy in these patients. We suggest that performing an extensive thrombophilia screen is indicated in COVID‐19 patients without conventional vascular risk factors who present with recurrent events concerning for thromboembolism.

## AUTHOR CONTRIBUTIONS

**SeyedehNarges Tabatabaee:** Conceptualization; writing – original draft. **Fatemeh Rezania:** Data curation; supervision; writing – review and editing. **Sevim Soleimani:** Resources; writing – original draft. **Zahra Mirzaasgari:** Supervision.

## CONFLICT OF INTEREST

The authors have no conflict of interest.

## CONSENT

Written informed consent was obtained from the patient to publish this report in accordance with the journal's patient consent policy.

## Data Availability

All data regarding the cases has been reported in the manuscript. Contact the corresponding author if you are interested in any further information.
